# Incompatibility of Lacrimal Syringing Test with Dacryoscintigraphy in Patients Undergoing Successful Dacryocystorhinostomy Surgery

**DOI:** 10.1055/s-0044-1788595

**Published:** 2024-08-16

**Authors:** Titap Yazicioglu, Elif Sarı, Selin Kesim

**Affiliations:** 1 Department of Ophthalmology, Kartal Dr. Lutfi Kirdar City Hospital, Istanbul, Türkiye; 2 Department of Nuclear Medicine, Kartal Dr. Lutfi Kirdar City Hospital, Istanbul, Türkiye

**Keywords:** epiphora, lacrimal drainage system, lacrimal scintigraphy, lacrimal syringing, nasolacrimal duct obstruction

## Abstract

**Purpose** The aim of our study was to evaluate the compatibility of lacrimal syringe test with dacryoscintigraphy (DSG) in the postoperative evaluation of external dacryocystorhinostomy (Ext-DCR) surgery.

**Material and Methods** Thirty eyes of 30 patients suffering from unilateral epiphora with discharge and showing complete obstruction on lacrimal irrigation test were included in the study. Patients with dysfunctional lacrimal pump function, revision surgery, partial obstruction, and ocular surface diseases were not included in the study.

Verification of the nasolacrimal duct obstruction (NLDO) was achieved with dye disappearance test, Jones test 1 (JT 1), and JT 2. According to the type of obstruction seen on DSG, patients were classified into two groups: presac and postsac obstruction. Patients with complete obstruction detected in the lacrimal syringing and dynamic scintigraphy underwent Ext-DCR, and the results were evaluated.

**Results** Thirty patients, mean age 58.93 ± 12.11 years, all with unilateral NLDO were included in the study. All had grade 5 Munk score epiphora and discharge. The mean duration of obstruction was 24.57 ± 10.65 months. In the lacrimal irrigation test, all patients had complete obstruction in one eye, while the other eye was normal. According to preoperative DSG results, there were 20 (66.7%) patients with presac delay and 10 (33.3%) patients with postsac delay. All patients underwent Ext-DCR with silicone tube intubation and were followed for 1 year. Although there was symptomatic improvement in all patients and the lacrimal syringing test was patent, no change was seen in scintigraphy.

**Conclusion** Although DSG is a sensitive nuclear medicine method, it is not useful for predicting the functional success of the Ext-DSR.

## Introduction

Primary acquired nasolacrimal duct (NLD) obstruction (PANDO) is a common cause of epiphora in adults and dacryocystorhinostomy (DCR) surgery is performed to control epiphora and achieve functional restoration of the lacrimal system. 1 In cases of partial or complete obstruction of the NLD, deciding whether patients will benefit from surgery is often based on a combination of clinical examination. 2 Objective methods such as irrigation and dacryoscintigraphy (DSG) are the mainstay to evaluate the results of this treatment. In clinical practice, lacrimal syringing is the most commonly performed test to investigate epiphora, providing valuable information about the presence, location, and nature of lacrimal pathway. 3 It helps to evaluate whether the canaliculus, lacrimal sac, and NLD are qualitatively patent, partially or completely obstructed. Assessing subjective control of complaints is also important to provide greater reability. 1 3

Verification of the NLD obstruction (NLDO) is achieved using passive and active investigating procedures. Commonly used tests to evaluate the lacrimal drainage system are dye disappearance test (DDT), Jones test 1 (JT 1), and Jones test 2(JT 2). 4

DSG is a sensitive and noninvasive imaging modality used in the evaluation of drainage of tears, and was first introduced by Rossomondo et al in 1972, and is widely used in the diagnosis of lacrimal system abnormalities. 5 6 It is a radionuclide procedure that shows the passage of radioactive agent with tear from the conjunctival sac to the nasal cavity through the ampulla, canaliculi, lacrimal sac, NLD, and nasal cavity, and allows the evaluation of lacrimal pump function and tear drainage. 7 8 The main indications of scintigraphy are evaluation of epiphora, detection of subclinical lacrimal duct obstruction, selection of suitable patients for surgery, and evaluation of DCR success. It is contraindicated in acute infective and allergic conditions of the eye. 9

The aim of this study was to investigate whether lacrimal scintigraphy can be a good predictor in the postoperative evaluation of patients who underwent successful external DCR (Ext-DCR) due to NLDO and whose postop lacrimal irrigation test was patent and found anatomically (objectively) and functionally (subjectively) successful.

## Material and Methods

Thirty patients of 30 eyes with unilateral NLDO (mean age, 58.93 ± 12.11 years) (range, 40–78 years, 7 men, 23 women) who were complaining about epiphora with mucopurulent discharge, between September 2023 and March 2024, were included in this study.

The study protocol adhered to the tenets of Declaration of Helsinki and was approved by the ethics committee. Fully informed written consent form was taken from all patients.

Patients' demographic information, visual acuity, clinical history, details of onset, severity, and frequency of watering were taken and endoscopic examination was performed to evaluate whether there was any nasal pathology. Ocular surface examination, the location and size of the punctum, laxity of the eyelid, or paralysis of the orbicularis oculi were observed. Patients with dysfunctional lacrimal pump function due to laxity of eyelids or paralysis of the orbicularis oculi muscle, canalicular injury, revision surgery, partial obstruction, and ocular surface diseases (keratitis, conjunctivitis) affecting tear secretion were not included in the study. Complications encountered during the follow-up period were all noted.

To confirm a stenosis of the outflow tract, diagnostic probing and syringing were performed in all patients with thickened, increased tear lake and reflux on expression of the lacrimal sac. Verification of the NLDO was achieved with DDT, JT 1, and JT 2. After a drop of 2% fluorescein is instilled in the fornix, the fluorescein remaining in the cul-de-sac is examined and the amount of fluorescein graded using a scale from 0 to 4, where 0 indicates no remaining dye. The JT 1 and JT 2 assess the flow of tears into the lacrimal sac and through the NLD. The presence of dye in the inferior meatus indicates a positive result of Jones test.

The lacrimal system was regarded as freely patent if there was no regurgitation at the punctum. Complete obstruction was considered if the serum completely refluxed and did not irrigate into the nose; partial obstruction was characterized by partial irrigation into the nose with some extent of reflux. DSG was subsequently performed on a different day, and the degree of obstruction was classified into patent, partial obstruction, and complete obstruction. Patients with complete obstruction were included in the study.

Radionuclide scintigraphy was done with patient sitting upright in front of the pinhole collimator of a gamma camera. Before instillation of radionuclide, manual pressure over the inner canthus empties the lacrimal sac. A drop of techetium-99m pertechnetate of 1.85 to 3.7 MBq (50–100 µCi) was placed in the lateral conjunctival sac using a micropipette. The patient was asked to remain in position and blink normally. Dynamic imaging was started immediately and continued every minute for the first 10 minutes, followed by static imaging at 11, 40, and 60 minutes. Patients were classified into two groups (presac and postsac obstruction) under the obstruction type of lacrimal scintigraphy.

Ext-DSG was performed in patients with complete obstruction, and postoperative DSG was performed for at least 6 months after the surgery to evaluate the patency of the lacrimal system. The primary outcome was assessed by surgical success, defined both as anatomical (objective) and functional (subjective). Anatomical success was defined as endoscopic dye visualization of the nasal opening of the anastomosis, functional success was defined as complete resolution of epiphora (Munk score grade 0) or improvement of epiphora (Munk score grade 1).

## Results

A total of 30 patients with epiphora were included in the study. There were 23 (76.7%) female and 7 (23.3%) male with a mean age of 58.93 ± 12.11 years (range, 40–77 years). There were 20 (66.7%) right and 10 (33.3%) left eye involvement. The mean duration of obstruction was 24.57 ± 10.65 months (range, 12–50 months). In the lacrimal irrigation test, all patients had complete obstruction in one eye, while the other eye was normal. Demographics of enrolled patients are shown in Table 1 .

**Table 1:** Patients demographics information

Gender	
Male/Female	7/23
Age	
Mean	58.93 ± 12.11 y
Range	40–78 y
Laterality	
Right/Left	20/10
No. of complete obstruction	30
Duration of symptoms	
Mean	24.57 ± 10.65 mo
Range	12–50 mo

All patients had thickened, increased tear lake and reflux on expression of the lacrimal sac, with a Munk score of grade 5, grade 4 DDT test, and negative JT, but the results of all these tests improved after the surgery. According to preoperative DSG results, there were 20 (66.7%) patients with presac delay and 10 (33.3%) patients with postsac delay ( Table 2 ).

**Table 2:** Patients' clinical information and pre- and postoperative test results

Dacryoscintigraphy type	
Presac obstruction	20 (66.7%)
Postsac obstruction	10 (33.3%)
Investigating procedures	
Fluorescent dye disappearance test (DDT)	
Preop	Grade 4
Postop	Grade 0
Jones test 1	
Preop	Negative
Postop	Positive
Jones test 2	
Preop	Negative
Postop	Positive
Munk score	
Preop	Grade 5
Postop	Grade 0–1
External dacryocystorhinostomy	
Anatomical success	30 (100%)
Functional success	30 (100%)

**Fig. 1:**
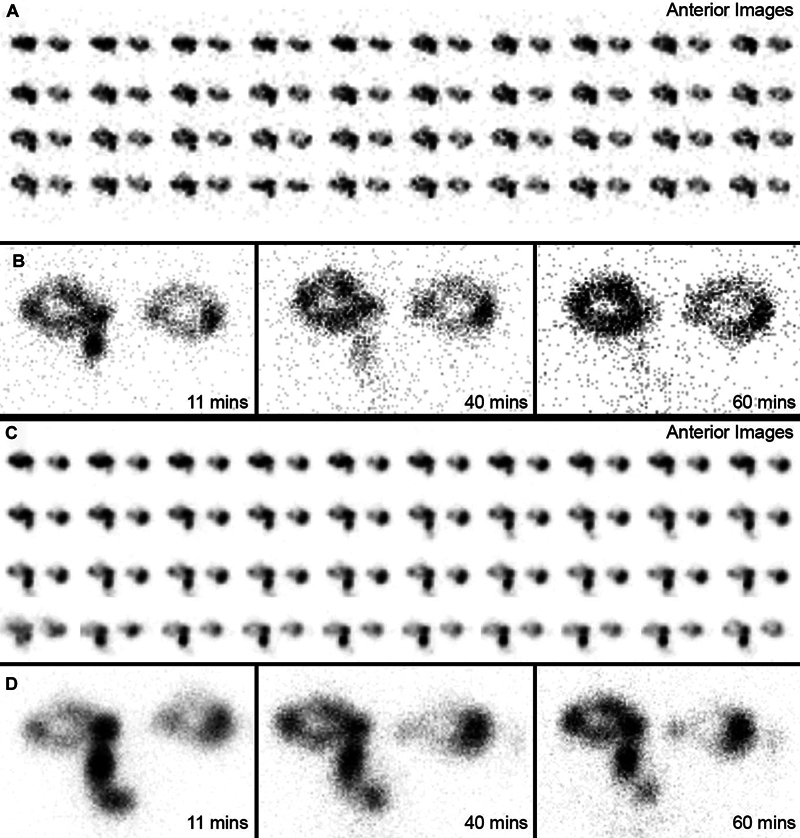
( **A** ) Preoperative immediate dynamic, ( **B** ) early and late static images of dacryoscintigraphy show unilateral obstruction at the left presac level. Postoperative dacryoscintigrams reveal no change ( **C** , **D** ).

**Fig. 2:**
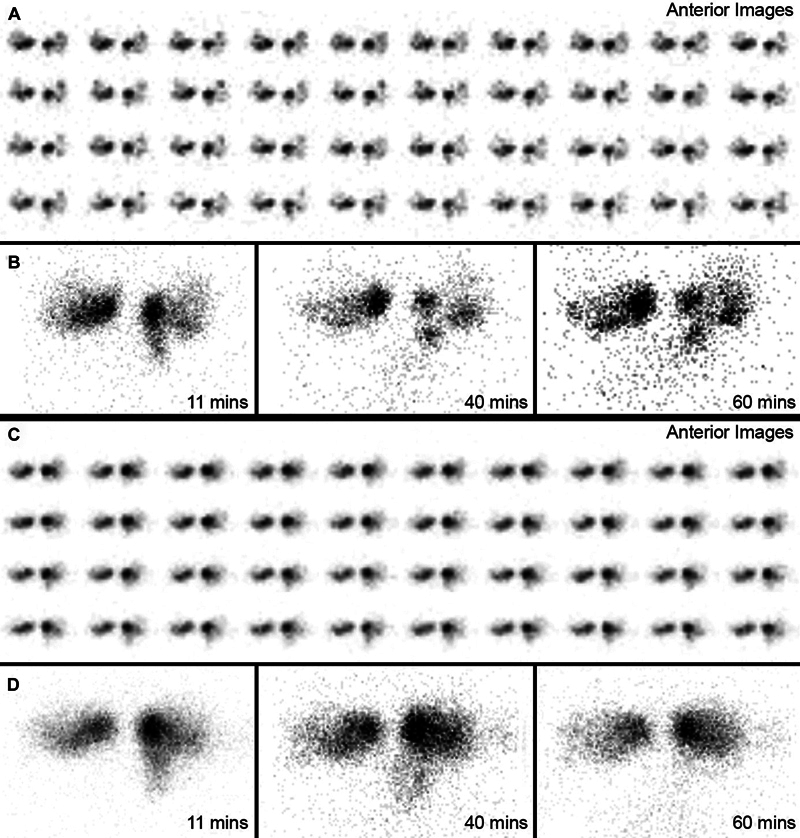
( **A** ) Preoperative immediate dynamic, ( **B** ) early and late static images of dacryoscintigraphy show unilateral obstruction at the postsac level. Postoperative dacryoscintigrams reveal no change ( **C** , **D** ).

All patients had Ext-DCR with silicone tube intubation, and were followed for 1 year. Postoperatively, lacrimal irrigation test showed patency but DSG showed total obstruction in all operated patients ( Figs. 1 and 2 ).

## Discussion

In NLDO, deciding whether patients will benefit from surgery is usually based on clinical examination. Evaluation of the drainage system by slit lamp examination for patency of the punctum and syringing of the lacrimal sac provides valuable information about the presence, location, and nature of the obstruction. 2 10 If there is reflux from the punctum opposite the injection side suggests an obstruction in the common canaliculus or more distally, while direct reflux of saline from the injected punctum and failure to advance the cannula into the lacrimal fossa suggests complete obstruction of the canaliculus. In addition, when serous reflux material is seen, it indicates the presence of canalicular, common canalicular, or upper lacrimal sac obstruction, and when viscous reflux material is seen, it indicates the presence of obstruction in the distal region such as lacrimal sac or NLD. 3

Confirmation of NLDO is achieved using investigative procedures. The Jones test and DDT are subjective tests that roughly describe the function of the lacrimal system and do not provide accurate information about the physiological status or anatomical integrity of the lacrimal passage. 11

One of the imaging techniques used in objective evaluation of the lacrimal drainage system is radionuclide scintigraphy. It is a safe, noninvasive, low-radiation-dose nuclear medicine method and increasingly used to evaluate the function of the lacrimal drainage system. 11 12 It was stated that with this functional and morphological method, the localization of lacrimal drainage system (LDS) obstructions can be distinguished and accordingly positive scintigrams the location of the obstruction can be divided into subgroups as prelacrimal sac, lacrimal sac/duct junction, and duct obstructions. 12 13

DSG was performed to evaluate postoperative epiphora and concluded that this simple and easy-to-perform procedure helps detect subclinical and partial lacrimal duct obstruction with good patient compliance. 10 14 Moreover, in some clinics measurement is routinely performed with quantitative DSG, which permits to quantify tear clearance rate, to determine the transit time taken for tracer material to reach the lacrimal sac and the nasal cavity. 10 12 15 In one of the studies where transit time (T1) and linear clearance rate (LCR) were measured with quantitative DSG, it was stated that T1 was found significantly higher before DCR surgery and LCR was significantly lower than values obtained from controls. Significant improvement was observed in both scintigraphic values in the postoperative patient group, underlining the success of the surgery. 14

In one of the studies comparing lacrimal scintigraphy of patients undergoing Ex-DCR surgery with a healthy control group showed significant delay in tear drainage. It was stated that cicatricial changes occurring around the sac after removal of the medial wall of the sac negatively affect the lacrimal pump mechanism. 15

In our study, all patients had grade 5 Munk score of epiphora and discharge. Lacrimal irrigation test showed complete obstruction in one eye, while the other eye was normal. Patients had undergone Ext-DCR with silicone tube intubation. Although symptoms of epiphora resolved and the lacrimal syringing test showed patency of NLD in all operated eyes, lacrimal scintigraphy showed abnormal for delayed appearance of tracer material. Our results show that although care is taken to preserve the medial canthal ligament in Ext-DCR, the lacrimal pump mechanism is affected by DCR and affects tear kinetics.

It was stated that although DSG is widely used to evaluate the diagnosis of abnormalities, it can only provide planar images and therefore needs to be improved. 12

In our study, it was surprising that although there was an improvement in the epiphora complaints of the patients, no change was observed in the pre- and postoperative scintigraphy.

A limitation of our study is that we did not compare postoperative documentation of NLD with other imaging techniques.

## Conclusion

Ext-DCR is effective enough to relieve epiphora complaints of PANDO with a high success rate, but the dynamic function of the drainage system remains lower than a normal system. We think that lacrimal scintigraphy is not suitable for the postoperative evaluation of NLD as it does not correlate with the symptoms and clinical findings.
